# Evaluation of pharmaceutical intervention in direct-acting antiviral agents for hepatitis C virus infected patients in an ambulatory setting: a retrospective analysis

**DOI:** 10.1186/s40780-018-0113-3

**Published:** 2018-07-17

**Authors:** Haruna Yamamoto, Hiroaki Ikesue, Mai Ikemura, Rieko Miura, Kazumi Fujita, Hobyung Chung, Yoshiki Suginoshita, Tetsuro Inokuma, Tohru Hashida

**Affiliations:** 1 Department of Pharmacy, Kobe City Medical Center General Hospital, 2-1-1 Minatojima Minamimachi, Chuo-ku, Kobe, Hyogo 650-0047 Japan; 2 Department of Clinical Pharmacy, Faculty of Pharmaceutical Sciences, Kobe Gakuin University, 1-1-3 Minatojima, Chuo-ku, Kobe, 650-8586 Japan; 3 Department of Gastroenterology and Hepatology, Kobe City Medical Center General Hospital, 2-1-1 Minatojima Minamimachi, Chuo-ku, Kobe, 650-0047 Japan

**Keywords:** Hepatitis C virus, Direct-acting antivirals, Pharmacist, Ambulatory care, Outpatients, Adherence, Sustained virological response

## Abstract

**Background:**

Direct-acting antivirals (DAAs) are known to improve tolerability and have higher efficacy and shorter treatment durations compared with conventional interferon (IFN)-based treatments for hepatitis C virus (HCV) infection. Management of drug interactions and maintenance of patient adherence are important to achieve adequate therapeutic effects, sustained virological response (SVR). In order to maximize the benefits of oral DAA therapy, we established an ambulatory care pharmacy practice, a model of integrated collaboration between physicians and pharmacists, for patients receiving IFN-free DAA therapy. In this study, we evaluated pharmaceutical intervention for patients visiting the ambulatory care pharmacy practice.

**Methods:**

HCV-infected outpatients who visited our ambulatory care pharmacy practice between September 2014 and May 2017 were eligible for inclusion in the study. When IFN-free DAAs were first prescribed, the physicians recommended all patients to visit the ambulatory care pharmacy practice after their clinical examination. Subsequently, at the second visit or later, the patients visited the pharmacy service before the physician’s examination. The primary endpoint was SVR, defined as HCV RNA below the lower limit of quantification after the completion of treatment. We also evaluated the adherence rate to DAAs, suggestions to the physicians by the pharmacists, and questions from the patients. All data were obtained retrospectively using an electronic medical record system.

**Results:**

Among the 401 study subjects, 386 patients completed the IFN-free DAA therapy. A total of 365 patients have reached 12 or 24 weeks after completing the treatment. The overall SVR rate was 98.1% (358/365). The proportion of patients with adherence ≥90% was 99.3% (398/401). Two-hundred and sixty-seven (84%) among 318 suggestions of prescription made by the pharmacists mainly to manage the adverse events were accepted by the physicians. The pharmacists received and answered 1072 questions on DAA therapy from the patients.

**Conclusions:**

This study indicates that the pharmaceutical intervention may contribute to enhanced adherence to DAAs and higher SVR rates in comparison with previous reports. This study also demonstrates that collaboration between physicians and pharmacists in an ambulatory setting provides favorable outcomes for patients receiving IFN-free DAAs.

## Background

Chronic hepatitis C is caused by the infection of hepatitis C virus (HCV) and approximately 185 million are infected worldwide [1, 2]. Chronic hepatitis C can develop liver cirrhosis over time, followed by hepatocellular carcinoma, which is a life-threatening disease. Thus, eradication of HCV is important to prevent progression to hepatocellular carcinoma. In the past, interferon (IFN)-based regimens constituted the major therapeutic approaches to the treatment of chronic hepatitis C. However, there are some problems, including insufficient therapeutic effect and various types of severe adverse drug events.

Since 2014, several IFN-free regimens, based on combinations of direct-acting antivirals (DAAs), have been launched for the treatment of HCV infection in Japan. These have some attractive characteristics, including oral medication, fewer adverse drug events, higher efficacy, and shorter treatment durations, compared with conventional IFN-based therapy [3–8]. Meanwhile, DAAs have some interactions with some drugs, caused by induction or inhibition of cytochrome P450 or pH changes in the stomach. The adherence of these agents can reflect the efficacy of DAA therapy [5, 6, 9]. Therefore, management of drug interactions and facilitating patient adherence to DAAs are important to achieve adequate therapeutic effects.

In order to maximize the benefits of oral DAA therapy, the physicians in our hospital asked the pharmacists to provide a consultation service, in addition to the conventional physician’s examination. We established an ambulatory care pharmacy practice, a model of integrated collaboration between physicians and pharmacists, following the introduction of IFN-free DAA therapy in our hospital. In the ambulatory care pharmacy practice, the pharmacists help to facilitate the IFN-free DAA therapy through the patients’ education and counseling. There is little information regarding collaborative care models for the successful treatment of patients receiving DAA therapy. In this study, we evaluated the clinical impact and role of the pharmaceutical intervention in DAA therapies for HCV infected patients in an ambulatory care setting.

## Methods

### Establishment of an ambulatory care pharmacy practice for HCV-infected outpatients receiving DAA therapy

We established an ambulatory care pharmacy practice for HCV-infected outpatients receiving IFN-free DAA therapy in September 2014, when we started therapy with DAAs in Kobe City Medical Center General Hospital. The purposes of establishing this practice were to provide information to physicians for appropriate prescriptions and to patients for the resolution of unclear points, to minimize the potential influence of drug-drug and drug-food interactions, to prevent adverse drug events, to enhance the patients’ adherence, and to maximize the effectiveness of IFN-free DAA therapy through patient education and counseling on DAA treatments [10, 11]. The DAA therapy includes daclatasvir tablets and asunaprevir capsules (DCV + ASV), sofosbuvir/ledipasvir combination tablets (SOF/LDV), ombitasvir/paritaprevir/ritonavir combination tablets (OBV/PTV/r), and elbasvir tablets and grazoprevir tablets (EBR + GZR) for HCV genotype 1-infected patients and sofosbuvir tablets and ribavirin tablets or capsules (SOF + RBV), and OBV/PTV/r tablets and ribavirin capsules (OBV/PTV/r + RBV) for HCV genotype 2-infected patients. The standard duration of the therapy is 24 weeks for DCV + ASV, 16 weeks for OBV/PTV/r + RBV, and 12 weeks for the other combinations. The ambulatory care pharmacy practice consisted of four pharmacists, with 4, 6, 9, and 19 years of experience as a hospital pharmacist, respectively.

**Fig. 1:**
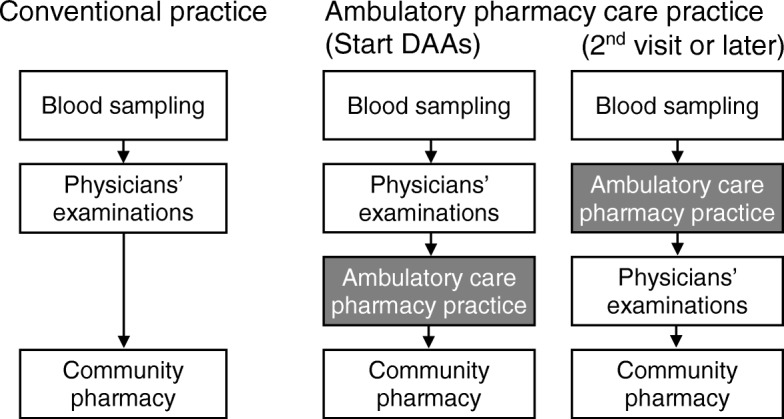
Patient flow in the conventional and established ambulatory care pharmacy practice. When IFN-free direct-acting antivirals (DAAs) were first prescribed, all patients visited the ambulatory care pharmacy practice after being examined by a physician. The pharmacist provided patient education, advised on the timing of taking DAAs, based on the patients’ life styles, enquired regarding concomitant medications and supplements for the prevention of drug interaction, and suggested that the physicians change the concomitant medications as necessary. At the second visit or later, the patients visited the pharmacy service before consulting the physician. The pharmacist had a face-to-face consultation with each patient and suggested modifications of the plan of treatment to the physician, based on the patient’s condition

A patient flow chart, including the ambulatory care pharmacy practice, is shown in Fig. 1. When IFN-free DAAs were first prescribed, the physicians advised all patients to visit the ambulatory care pharmacy practice after their clinical examination and called to a pharmacist. The pharmacist explained the following points to the patients at their first visit: (1) the duration of the treatment with DAAs, (2) the importance of adherence for successful treatment, (3) recommendation of the time to take the DAAs, based on the patient’s lifestyle and concomitant medications, (4) appropriate management following a missed dose, (5) adverse drug events from DAAs, and (6) drug-drug and drug-food interactions. The pharmacist also asked about concomitant medications and supplements in order to avoid drug interaction and suggested the physicians to change the concomitant medications as necessary. At the second visit or later, the patients visited the pharmacy service before a physicians’ examination. The pharmacist checked (1) the adverse drug events, (2) changes to any concomitant medications or supplements, (3) adherence to DAAs by counting the empty pill sheets or one-dose packages of DAAs, and (4) asked whether the therapy had been provided without any problems. Patients were able to ask a pharmacist questions directly at the ambulatory care pharmacy practice, or by telephone. Pharmacists who received questions from patients shared the information with the physicians through face-to-face meetings and/or by telephone and through the electronic medical record system [12]. As necessary, the pharmacist suggested the prescriptions to the physicians, based on the information obtained. After the prescriptions were verified by the pharmacists, the patients received the DAAs at community pharmacies.

### Patients

Participants were eligible for inclusion if they were HCV-infected outpatients who visited our ambulatory care pharmacy practice between September 3, 2014 and May 31, 2017. They were excluded if they were younger than 20 years old or their therapeutic process was unknown.

### Outcome measures and data collection

The primary endpoint in this study was a sustained virological response (SVR) defined as HCV RNA below the lower limit of quantification after the completion of treatment. SVR was evaluated at 24 weeks after the completion (SVR24) only in patients treated with DCV + ASV and at 12 weeks (SVR12) in those treated with other regimens [13]. SVR was evaluated only in those patients who had reached 24 or 12 weeks after treatments at the time of data cutoff (September 30, 2017). As baseline characteristics of patients, we evaluated sex, age, the presence or absence of cirrhosis, previous treatment for HCV, genotype of HCV, therapeutic agents for HCV, and hematological tests (serum HCV RNA, albumin, total bilirubin, aspartate aminotransferase (AST), alanine transferase (ALT), number of platelets, prothrombin time, and hemoglobin). For the patients with genotype 1 HCV, the nonstructural protein 5A (NS5A) resistance associated variants L31/Y93 were also evaluated.

We also evaluated the adherence rate, relative dose intensity (RDI), and the suggestions to the physicians by the pharmacists, questions from the patients in the ambulatory care pharmacy practice. The adherence was evaluated by counting the number of the empty pill sheets or one-dose packages of DAAs at 2, 4, 8, 12 (all of DAAs), 16 (OBV/PTV/r + RBV and DCV + ASV) and 24 (DCV + ASV) weeks after the initial treatment at the ambulatory care pharmacy practice. Initially, SOF/LDV and SOF were supplied in a bottle containing 28 tablets, which was changed to a press through packages (PTP) sheet after March 2017. Thus, until March 2017, physicians in our hospital prescribed those medicines as one-dose packages and requested the community pharmacy to print consecutive dates for taking the pills on each one-dose package. To obtain accurate adherence data, we asked patients to bring the empty packages at each visit to the ambulatory care pharmacy practice. Adherence to DAAs was recorded by pharmacists by counting the number of empty packages or the empty PTP sheets of medicine. The adherence rate was calculated as follows: [the number of tablets or capsules taken] / [the number of tablets or capsules prescribed] × 100 (%). RDI was calculated as follows: [the number of tablets or capsules taken] / [the number of tablets or capsules planned] × 100 (%) [14]. Each investigation item was obtained retrospectively using the electronic medical record system.

### Ethics

This study was approved by the Institutional Review Board of Kobe City Medical Center General Hospital and the Board waived the need for patients’ consent (No. zn170408).

## Results

### Baseline characteristics of patients

**Fig. 2:**
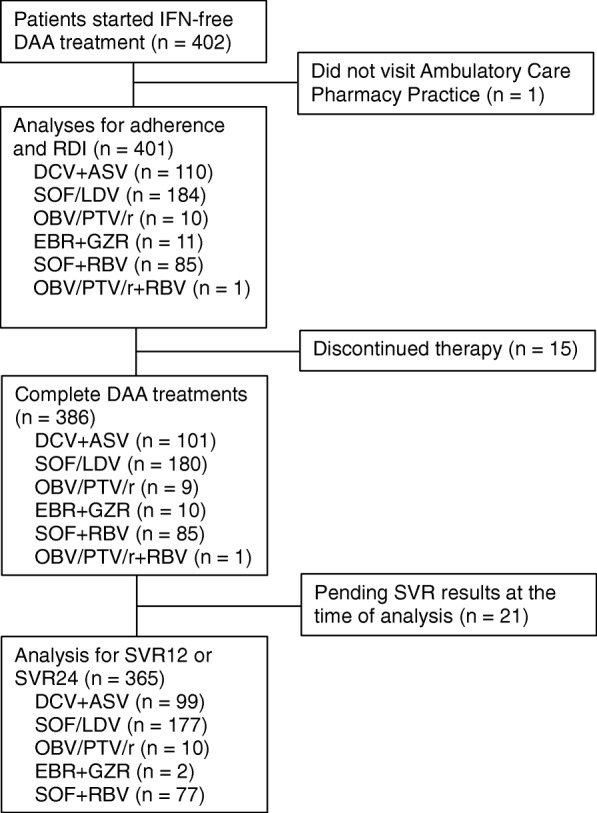
Flow diagram of enrollment in study and analyses. The numbers of patients who were enrolled and analyzed in this study is described. The number of excluded patients and reasons for exclusion are also described. IFN, interferon; DAA, direct-acting antiviral agent; RDI, relative dose intensity; DCV, daclatasvir; ASV, asunaprevir; SOF, sofosbuvir; RBV, ribavirin; LDV, ledipasvir; OBV, ombitasvir; PTV, paritaprevir; r, ritonavir; EBR, elbasvir; GZR, grazoprevir and SVR; sustained virological response

A total of 402 patients visited our ambulatory care pharmacy practice. Only one patient who never visited was excluded from this study. The flow diagram of study enrollment and the number of patients meeting the exclusion criteria for analysis is shown in Fig. 2. The baseline characteristics of the 401 study patients are summarized in Table 1. The numbers of patients with HCV genotypes 1 and 2 were 315 (78.6%) and 86 (21.4%), respectively. Of the patients with HCV genotype 2, 51 and 35 patients were infected with HCV subtype 2a and 2b, respectively. The patients received DCV + ASV (*n* = 110, 27.4%), SOF/LDV (*n* = 184, 45.9%), OBV/PTV/r (*n* = 10, 2.5%), EBR + GZR (*n* = 11, 2.8%), SOF + RBV (*n* = 85, 21.2%), or OBV/PTV/r + RBV (*n* = 1, 0.2%).

**Table 1:** Baseline characteristics of the patients

Characteristics	Overall (n = 401)	Genotype 1 (n = 315)	Genotype 2 (n = 86 )
Male sex, n (%)	170 (42.4%)	132 (41.9%)	38 (44.2%)
Age, median years (range)	67 (22–87)	68 (31–87)	65 (22–84)
Cirrhosis, n (%)	89 (22.2%)	75 (23.8%)	14 (16.3%)
Treatment experienced, n (%)	123 (30.7%)	110 (34.9%)	13 (15.1%)
HCV RNA, log IU/mL, median (range)	6.1 (2.0–7.5)	6.1 (2.0–7.5)	5.9 (3.0–7.2)
Albumin, g/dL, median (range)	4.1 (2.6–4.9)	4.1 (2.6–4.9)	4.1 (2.9–4.8)
Total bilirubin, mg/dL, median (range)	0.7 (0.2–28.1)	0.7 (0.2–28.1)	0.7 (0.2–2.4)
AST, IU/L, median (range)	41 (10–360)	42 (10–360)	38 (14–250)
ALT, IU/L, median (range)	42 (5–382)	42 (8–382)	40 (5–251)
Platelets, ×10^4^/mm^3^, median (range)	14.6 (0.6–55.2)	14.6 (0.6–55.2)	14.4 (4.0–30.0)
Prothrombin time, %, median (range)	90.0 (16.3–134.2)	90.3 (20.4–134.2)	86.9 (16.3–121.1)
Hemoglobin, g/dL, median (range)	13.4 (1.1–18.4)	13.4 (1.1–17.0)	13.5 (9.1–18.4)
NS5A resistance associated variants^a^, n (%)			
Neither L31 or Y93	–	184 (58.4%)	–
Either L31 or Y93	–	40 (12.7%)	–
Both L31 and Y93	–	2 (0.6%)	–
Treatment agents, n (%)			
DCV+ASV	110 (27.4%)	110 (34.9%)	0 ( 0%)
SOF/LDV	184 (45.9%)	184 (58.4%)	0 ( 0%)
OBV/PTV/r	10 ( 2.5%)	10 ( 3.2%)	0 ( 0%)
EBR+GZR	11 ( 2.8%)	11 ( 3.5%)	0 ( 0%)
SOF+RBV	85 (21.2%)	0 ( 0%)	85 (98.8%)
OBV/PTV/r+RBV	1 ( 0.2%)	0 ( 0%)	1 ( 1.2%)

^a^Eighty-nine patients treated with SOF/LDV were not checked

*AST* Aspartate aminotransferase, *ALT* Alanine aminotransferase, *NS5A* Nonstructural protein 5A, *DCV* Daclatasvir, *ASV* Asunaprevir, *SOF* Sofosbuvir, *LDV* Ledipasvir, *OBV* Ombitasvir, *PTV* Paritaprevir, *r* Ritonavir, *EBR* Elbasvir, *GZR* Grazoprevir and *RBV* Ribavirin

### Virological response to IFN-free DAA therapy

Among the 401 study patients, 365 reached 12 or 24 weeks after completing the DAA treatment. Ninety-nine patients treated with DCV + ASV and 266 patients treated with the other agents had reached 24 and 12 weeks after the completion, respectively. Ninety-three of the 99 patients (93.9%) treated with DCV + ASV achieved SVR24, and 265 of 266 patients (99.6%) who received the other agents achieved SVR12 (Table 2). One patient treated with SOF/LDV had a relapse of HCV, and two patients treated with DCV + ASV had virological breakthrough.

**Table 2:** Rates of patient sustained virological responses and adherence to DAA treatments

Outcomes	DCV+ASV (n = 110)	SOF/LDV (n = 184)	OBV/PTV/r (n = 10)	EBR+GZR (n = 11)	SOF+RBV (n = 85)	OBV/PTV/r +RBV (n = 1)	TOTAL (n = 401)
Adherence							
100%, n (%)	84 (76.4%)	170 (92.4%)	10 (100%)	10 (90.9%)	65 (76.5%)	0 (0%)	339 (84.5%)
≥95%, n (%)	109 (99.1%)	182 (98.9%)	10 (100%)	10 (90.9%)	85 (100%)	1 (100%)	397 (99.0%)
90–94%, n (%)	0 (0%)	1 (0.5%)	0 (0%)	0 (0%)	0 (0%)	0 (0%)	1 (0.2%)
<90%, n (%)	1 (0.9%)	1 (0.5%)	0 (0%)	1 (9.1%)	0 (0%)	0 (0%)	3 (0.7%)
Completion the treatments, n (%)	101	180	9	10	85	1	386
Achieved SVR12, n (%) ^a^	–	176/177 (99.4%)	10/10 (100%)	2/2 (100%)	77/77 (100%)	0/0 (0%)	265/266 (99.6%)
Achieved SVR24, n (%) ^a^	93/99 (93.9%)	–	–	–	–	–	93/99 (93.9%)

^a^Patients were excluded if they have not reached12 or 24 weeks after the completion of treatment

*DCV* Daclatasvir, *ASV* Asunaprevir, *SOF* Sofosbuvir, *RBV* Ribavirin, *LDV* Ledipasvir, *OBV* Ombitasvir, *PTV* Paritaprevir, *r* Ritonavir, *EBR* Elbasvir and *GZR* Grazoprevir

### Adherence to IFN-free DAA therapy

Among the 401 study patients, 386 (96.3%) completed the IFN-free DAA therapy, while 15 discontinued at an early stage of the therapy. The reasons for discontinuation were adverse drug events (*n* = 7, 46.7%), poor adherence (*n* = 4, 26.7%), virological breakthrough (*n* = 2, 13.3%), and exacerbation of concomitant diseases (n = 2, 13.3%). No patient died or experienced serious adverse drug events in IFN-free DAA therapy. Among 86 patients who received RBV (SOF + RBV or OBV/PTV/r + RBV), the RBV dose was reduced in 37 patients due to anemia or for other reasons.

We evaluated the adherence (Table 2) and RDI rate in the 401 patients. The numbers of patients with 100% adherence and RDI were 339 (84.5%) and 319 (79.6%), respectively. The numbers with ≥90% adherence and RDI were 398 (99.3%) and 374 (93.3%), respectively. The percentage of patients with ≥90% adherence for combination tablets and non-combination tablets was 99.5% (193/194) and 99.0% (205/207), respectively. There were no differences in the rates for the various regimens of IFN-free DAA therapy.

### Activities of pharmacists in the ambulatory care pharmacy practice

The pharmacists made a total of 318 suggestions to the physicians during the treatments (Table 3). Among these suggestions, the management of adverse drug events from DAAs (194 cases), including a prescription of analgesic agents for fever, and antiflatulent for diarrhea, were the most frequent. Of suggestions on drug-drug interactions (68 cases), the pharmacists suggested to change the timing of taking a histamine-2 receptor antagonist to avoid the interaction (18 cases). Of the 318 suggestions, 267 (84.0%) were accepted by the physicians.

**Table 3:** Numbers of suggestions provided by the pharmacists and the responses of the physicians

	Number of suggestions	Number of accepted suggestions by physicians (%)
At the beginning of the treatment		
Drug-drug interaction	68	44 (64.7%)
Dose reductions of ribavirin	6	6 (100%)
Second visit or later		
Management of adverse drug events	194	172 (88.7%)
Others	50	45 (90.0%)
TOTAL	318	267 (84.0%)

The pharmacists received 1072 questions about DAA therapy from the patients (Table 4). Questions about adverse drug events were the most common (577 cases, 53.8%), including rashes and pruritus. In addition, we also received some questions from community pharmacies. For example, when we received information from a community pharmacy that an arrival of DAA to the pharmacy would be delayed by several days, we discussed this with the prescribing physician and responded to the pharmacy to indicate that the delay in starting DAA treatments would be acceptable.

**Table 4:** Numbers of the questions from patients regarding DAAs treatment

Type of questions	Number (%)
Adverse drug events	577 (53.8%)
Drug interaction ^a^	205 (19.1%)
Management in the event of a missed dose of DAAs	57 ( 5.3%)
Others	233 (21.8%)
TOTAL	1,072 (100%)

*DAAs* Direct-acting antivirals

^a^ These included questions according to drug-drug (152 cases), drug-supplement (32 cases), and drug-food (21 cases) interactions

## Discussion

We have established an ambulatory care pharmacy practice for HCV-infected outpatients receiving IFN-free DAA therapy to maximize the benefits of the therapy. The pharmacists provided many suggestions to the physicians and a variety of information to the patients. In this study, we also evaluated the adherence and therapeutic effect of IFN-free DAA therapy. A total of 99.3% of the patients visiting our ambulatory care pharmacy practice achieved ≥90% adherence. The SVR rate was 99.6% in those patients who received DAAs other than DCV + ASV and 93.9% in those who received DCV + ASV.

The pharmacists received 1072 questions from the patients during the 33 months following the establishment of the ambulatory care pharmacy practice (Table 4). The majority of the questions were about adverse drug events and drug-drug interactions, which required pharmaceutical expertise for an adequate response. The pharmacists also provided 318 prescription suggestions and 84.0% of those suggestions were accepted by the physicians (Table 3). These results suggest that there is a significance of collaboration for IFN-free DAA therapy with physicians and pharmacists by taking advantages of each expertise.

Unlike in controlled clinical trials, the therapeutic effect of IFN-free DAA therapy in real-world clinical practice may be affected by lower adherence, some comorbidities and many concomitant medications. In phase 3 trials, patients with lower adherence to DAAs resulted in suboptimal SVR rates [5, 6]. Some studies have indicated that the SVR rates in adherent patients, defined as having the proportion of days covered (PDC) > 80% or 90%, were higher than in non-adherent [12, 15]. Enhancement of patients’ adherence to DAAs is a challenge for the achievement of satisfactory therapeutic effect of SVR through IFN-free DAA therapy in real-world clinical settings. The adherence to DAAs and SVR rates in previous reports are summarized in Table 5. In this study, 99.3 and 93.3% of patients achieved ≥90% adherence (Table 2) and RDI, a sufficiently high adherence to achieve the therapeutic effect. The adherence rate was not influenced by the dosage form (either the combination tablets or the non-combination tablets). Some previous reports have indicated that the SVR12 rate is 88.2–95.3% in real-world settings [9, 12, 16–18], and 84.4–98.1% in phase 2–3 trials [15, 19–21]. In this study, the SVR12 rate was 99.6% (Table 2), which is relatively higher than in the previous studies [9, 12, 16–18]. These results indicate that the pharmaceutical support provided by the ambulatory care pharmacy practice, including explanation of the importance of adherence and the management of adverse drug events, helped the achievement of favorable SVR rates, probably due to the enhancement of adherence.

**Table 5:** Comparison of the rate of SVR12 and adherence to DAAs

Reference (published year)	Study design	Genotype	Medications	Adherence assessment	SVR12 (%)	Adherence (%)
Present study	Retrospective	1 and 2	Various kinds of DAA	Pill count	99.6% (265/266)	Adherent (defined as ≥95% of pill count): 99.0% (397/401) Adherent (defined as ≥90% of pill count): 99.3% (398/401)
Retrospective analyses of clinical practice						
9 (2016)	Retrospective	1–3	Various kinds of DAA	Patient report	93.2% (124/133)	Adherent (defined as 100% of adherence): 79.1% (102/129)
12 (2017)	Retrospective	1–4	Various kinds of DAA	PDC from pharmacy record ^b^	Specialty pharmacy 92.9% (156/168)	Adherent (defined as ≥80% of PDC): 100% (206/206)
					Nonintegrated pharmacies 88.6% (39/44)	Adherent (defined as ≥80% of PDC): 97% (55/57)
16 (2016)	Retrospective	1 ^a^	SOF/LDV	PDC from pharmacy record ^b^	All subjects: 95.3% (928/974) Patients with adherence of ≥90%: 96.3% (621/645) <90%: 95.0% (301/317) <50%: 50.0% (6/12)	100% of PDC: 65.7% (632/962) ≥90% of PDC: 67.0% (645/962) ≥80% of PDC: 67.8% (653/962)
		2 or 3	SOF+RBV	PDC from pharmacy record ^b^	All subjects: 88.2% (585/663) Patients with adherence of ≥90%: 89.9%(561/624) <90%: 67.6% (23/34) <50%: 20.0% (1/5)	100% of PDC: 92.1% (606/658) ≥90% of PDC: 94.8% (624/658) ≥80% of PDC: 98.3% (647/658)
17 (2016)	Retrospective	1 ^a^	SOF, SOF/LDV, simeprevir	Patient report	90.4% (225/249)	Adherent (defined as 100% of adherence): 97.0% (360/371)
18 (2017)	Retrospective	1–3	Various kinds of DAA	PDC from pharmacy record ^b^	94.1% (350/372)	Mean PDC: 98.7% Adherent (defined as ≥95% of PDC): 98.9% (358/362)
Analyses of phase 2 and/or 3 clinical trials						
15 (2016)	Pooled analysis of phase 2-3 studies	1–6	SOF+RBV±LDV	Pill count	All subjects: 84.4% (2134/2528) Patients with adherence of ≥80%: 85.4% (1820/2131) <80%: 79.1% (314/397)	Mean: 91.3% Adherent (defined as ≥80% of doses): 84.3% (2131/2528)
	Pooled analysis of phase 2-3 studies	1–6	SOF/LDV	Pill count	All subjects: 96.5% (1441/1493) Patients with adherence of ≥80%: 97.1% (1394/1436) <80%: 82.5% (47/57)	Mean: 97.5% Adherent (defined as ≥80% of doses): 96.2% (1436/1493)
19 (2016)	Pooled analysis of phase 3 studies	1	SOF/ LDV±RBV	Pill count	96.7% (1888/1952)	Adherent (defined as ≥80% of doses): 92.3% (1802/1952)
20 (2016)	Pooled analysis of phase 3 studies	1–6	SOF/velpatasvir	Pill count	98.1% (1015/1035)	Adherent (defined as ≥90% of doses): 95.8% (992/1035)
21 (2016)	Phase 2a study	1	SOF/LDV	MEMS Pill counts Patient report	96.6% (58/60)	MEMS: mean 96.7% Pill counts: mean 98.2% Patient report: mean 99.3%

*MEMS* Electronic medication event monitoring system, *PDC* The proportion of days covered, *SOF* Sofosbuvir, *LDV* Ledipasvir, *RBV* Ribavirin

^a^The majority of the patients were infected with HCV genotype 1. Four patients were infected with genotype 4 and 175 patients were infected with an unknown genotype of HCV.

^b^PDC was defined as the total number of days with possession of medication in a period of time

There are some limitations in our study. First, there was no control group to allow a comparison of SVR and the adherence of our ambulatory care pharmacy practice, because we provided the service to all of our patients who received DAA therapy. Therefore, we estimated the usefulness of our pharmacy service by comparison with previous reports. Second, this is a single-center study and our findings might not be applicable to patient populations in other institutions. However, we speculate that the patient population in our institution is representative of Japanese patients. The third limitation is related with particular medical care system in Japan. The medical expense to receive DAA therapy is extremely low, 10,000 or 20,000 Japanese yen each month depending on the patient’s income, because of the public medical insurance system and the specific medical expenses subsidy system for viral hepatitis in Japan [22]. This system enabled the patients to receive DAA therapy much easier than other countries, while this might affect the results of our study.

## Conclusions

We established an ambulatory care pharmacy practice for HCV-infected outpatients receiving IFN-free DAA therapy to maximize the benefits of the therapy. The results of this study indicate that the pharmaceutical intervention may contribute to enhanced adherence to DAAs and higher SVR rates in comparison with previous reports. This study also demonstrates that an ambulatory care pharmacy practice, with collaboration between physicians and pharmacists, provides favorable outcomes for patients receiving IFN-free DAAs.

## Abbreviations

ALT: Alanine aminotransferase
AST: Aspartate aminotransferase
DAA: Direct-acting antivirals
DCV + ASV: Daclatasvir tablet and asunaprevir capsule
EBR + GZR: Elbasvir tablet and grazoprevir tablet
HCV: Hepatitis C virus
IFN: Interferon
NS5A: Nonstructural protein 5A
OBV/PTV/r: Ombitasvir/paritaprevir/ritonavir combination tablet
PDC: Proportion of days covered
RDI: Relative dose intensity
SOF + RBV: Sofosbuvir tablet and ribavirin tablet or capsule
SOF/LDV: Sofosbuvir/ledipasvir combination tablet
SVR: Sustained virological response
